# New insights into sperm rheotaxis, agglutination and bundle formation in Sharkasi chickens based on an in vitro study

**DOI:** 10.1038/s41598-022-17037-x

**Published:** 2022-07-29

**Authors:** Taymour M. El-Sherry, Hanan H. Abd-Elhafeez, M. A. M. Sayed

**Affiliations:** 1 Department of Theriogenology, Faculty of Veterinary Medicine, Assiut University, Assiut, 71526 Egypt; 2 Department of Cells and Tissues, Faculty of Veterinary Medicine, Assiut University, Assiut, 71526 Egypt; 3 Department of Poultry Production, Faculty of Agriculture, Assiut University, Assiut, 71526 Egypt

**Keywords:** Biological techniques, Cell biology

## Abstract

Fertility in birds is dependent on their ability to store adequate populations of viable sperm for extended durations in sperm storage tubules (SSTs). The exact mechanisms by which sperm enter, reside, and egress from the SSTs are still controversial. Sharkasi chicken sperm showed a high tendency to agglutinate, forming motile thread-like bundles comprising many cells. Since it is difficult to observe sperm motility and behavior inside the opaque oviduct, we employed a microfluidic device with a microchannel cross-section resembling close to that of sperm glands allowing for the study of sperm agglutination and motility behavior. This study discusses how sperm bundles are formed, how they move, and what role they may have in extending sperm residency inside the SSTs. We investigated sperm velocity and rheotaxis behavior when a fluid flow was generated inside a microfluidic channel by hydrostatic pressure (flow velocity = 33 µm/s). Spermatozoa tended to swim against the flow (positive rheotaxis) and sperm bundles had significantly lower velocity compared to lonesome sperm. Sperm bundles were observed to swim in a spiral-like motion and to grow in length and thickness as more lonesome sperm are recruited. Sperm bundles were observed approaching and adhering to the sidewalls of the microfluidic channels to avoid being swept with fluid flow velocity > 33 µm/s. Scanning and transmission electron microscopy revealed that sperm bundles were supported by a copious dense substance. The findings show the distinct motility of Sharkasi chicken sperm, as well as sperm's capacity to agglutinate and form motile bundles, which provides a better understanding of long-term sperm storage in the SSTs.

## Introduction

To achieve fertilization in humans and most animals, sperm and ovum must reach the fertilization site at the correct time. Therefore, copulation must take place prior to or during ovulation. Some mammalian species, such as dogs, and nonmammalian classes, such as insects, fish, reptiles, and birds, on the other hand, store sperm in their reproductive organs for long periods of time until their eggs are ready to be fertilized (asynchronous fertilization^1^). Birds are able to maintain viable sperm capable of fertilizing the ova for periods ranging from 2 to 10 weeks^2^.

This is a unique feature that distinguishes birds from other animals since it allows for a high probability of fertilization after a single insemination over several weeks without the need to synchronize mating and ovulation. The main sperm storage organ, called sperm storage tubules (SSTs), is located in the internal mucosal folds of the uterovaginal junction^3^. To date, the mechanisms of sperm entry, residency, and evacuation from sperm reservoirs are not fully understood. According to earlier investigations, there have been a number of hypotheses proposed for this, but none of them has been confirmed.

Forman^4^ proposed that sperm keep up their residency in the lumen of SSTs by continuous oscillatory movement against the direction of a fluid flowing through protein channels located on the epithelial cells of SSTs (rheotaxis). Because of the continual flagella activity required to keep sperm inside the SST lumen, ATP is depleted, and the motility is eventually decreased until the sperm are carried outside the sperm reservoirs by the fluid flow to begin a new journey by ascending the oviduct to fertilize the ovum (Forman^4^). This model for sperm storage is supported by the findings confirming the presence of aquaporines 2, 3, and 9 in the SSTs epithelium by immunocytochemistry^5^. To date, studies on sperm rheotaxis in chickens and the role it may play in SSTs storage, vaginal sperm selection, and sperm competition are lacking. In chickens, sperm ejaculate is deposited into the vagina following natural mating; however, more than 80% of the sperm are egressed from the vagina shortly after copulation^6^. This suggests that the vagina is the primary sperm selection site in avian species. Moreover, it has also been reported that less than 1% of sperm inseminate into the vagina enter the SSTs^2^. When chickens were inseminated artificially into the vagina, the number of sperm that reached the SSTs tended to increase, at 24 h post insemination. Until now, the mechanism of sperm selection in this process has been unknown, and the motility of sperm may play an important role in sperm uptake into SSTs^4^. It is difficult to monitor sperm motility in the oviduct directly in bird species due to the thick and opaque oviduct wall. Therefore, we lack basic knowledge of how sperm are transferred into SSTs after insemination.

Rheotaxis has recently been considered an important factor controlling sperm transport in mammalian genitalia^7^. Based on the ability of viable sperm to migrate against a flow, Zaferani et al.^8^ used a microfluidic corral system to passively isolate motile sperm from the semen sample inside a corral. This sperm sorting is required for medical infertility treatments and clinical studies and is favored compared to conventional methods which are time- and labor-consuming and could be detrimental to sperm morphology and structure integrity^8^. However, until now, there has been no study on the effect of flow in the genitalia of chickens on sperm motility.

Regardless of the mechanism that maintains sperm stored in SSTs, a number of researchers have observed that resident sperm agglutinate “head-to-head” forming bundles of agglutinated sperm in the SSTs of chickens^9,10^, quails^2^, and turkeys^11^. The authors suggested that a relationship exists between this agglutination and the extended period of sperm storage in the SSTs.

Tingari and Lake^12^, reported the close association between spermatozoa within the sperm-host glands of hens, and were curious as to whether avian spermatozoa agglutinated in the same way as mammalian sperm. They suggested that the deep connection between sperm cells in sperm glands could be due to pressure caused by the existence of large numbers of spermatozoa in a small space.

Transient evidence of agglutination was visible when spermatozoa behavior was evaluated in fresh hanging drop slides, notably along the edge of the semen droplet. However, the agglutination was frequently disrupted by the swirling action associated with continuous motility, explaining this phenomenon's fleeting nature^9^. The researchers also noted elongate "thread-like" cell aggregations when diluent was added to the semen.

An early attempt to simulate sperm glands was carried out by removing a delicate wire from a hanging drop slide, which caused an elongate vesicle to protrude from the semen droplet, similar to a sperm gland^9^. The sperm cells instantly lined up in a parallel pattern within the vesicle, but the overall unit quickly faded due to three-dimensional limitations. Consequently, in order to study sperm agglutinations, the need is to observe sperm motion and behavior directly in isolated sperm storage tubules and this is difficult to achieve. Therefore, devising a tool simulating the sperm gland is required to support studies of sperm motility and agglutination behavior. Brillard et al.^13^ reported that the average length of sperm storage tubules in mature chicken hens is 400–600 µm, but some SSTs can measure 2000 µm. Mero and Ogasawara^14^ classified sperm glands into enlarged and non-enlarged sperm storage tubules, both of which had similar lengths (about 500 µm) and neck widths (roughly 38 µm), but tubules of the two groups had average lumen diameters of 56.6 and 11.2 µm, respectively. In the current study, we used a microfluidic device with channel dimensions of 200 µm × 20 µm (W × H) to give a cross-section somewhat close to that of enlarged SSTs. In addition, we studied the sperm motility and agglutination behavior in a flowing fluid to be in line with Forman’s hypothesis that SSTs’ epithelial cells produce fluid that maintains sperm in the lumen via swimming against the flow direction (rheotaxis).

The objectives of the study were to overcome the problems of observing sperm motility inside the oviduct and to avoid the difficulties of studying sperm rheotaxis and behavior in a dynamic environment. A microfluidic device that generated hydrostatic pressure to model sperm motility inside the genitalia of chickens was used.

## **Results**

### Sperm velocity and rheotaxis (Table 2; Videos 1, 2)

When a drop of diluted semen sample (1:40) was loaded inside the microchannel device, two types of sperm motility (lonesome and bundles) were recognized. In addition, sperm showed a tendency to swim against the flow (positive rheotaxis; Videos 1, 2). Although sperm bundles had lower velocity than that of lonesome sperm (p < 0.001), they increased the percentage of sperm displaying positive rheotaxis (p < 0.001; Table 2). The percentage of positive rheotaxis was estimated to be approximately 53% and 85% for lonesome sperm and bundles, respectively.

### Characterization of bundle formation (Videos 3, 4) and figures (Figs. 2 and 3a,c)

The spermatozoa of Sharkasi chickens were observed to form thread-like bundles consisting of many tens of individuals immediately after ejaculation. These bundles increase in length and thickness with time and can remain in vitro for hours before dispersing (Video 3). These thread-like bundles are similar in shape to those of echidna sperm formed in the terminal segment of the epididymis. Sharkasi chicken sperm were found to have a high tendency to agglutinate and form a net of bundles less than a minute after collection. These bundles are motile and are capable of sticking to any adjacent wall or static object. Although sperm bundles reduce the velocity of sperm, it was obvious that they increase their linearity macroscopically. The bundles vary in length according to the number of sperm assembled in the bundle. Two segments of the bundle are recognized: the initial part, which comprises free heads of the agglutinated sperm, and the terminal part, including the tails and whole distal sperm. Using a high-speed camera (950 frames/s), we observed that the free heads of the agglutinated sperm, at the initial part of the bundle, are responsible for the bundle motility due to their oscillatory movements, which drag the rest of the bundle in a spiral-like movement (Video 4). However, in long bundles, some free heads of adhered sperm in the body and the terminal part of the bundle were observed to act like paddles and help in bundle movement.

**Figure 1:**
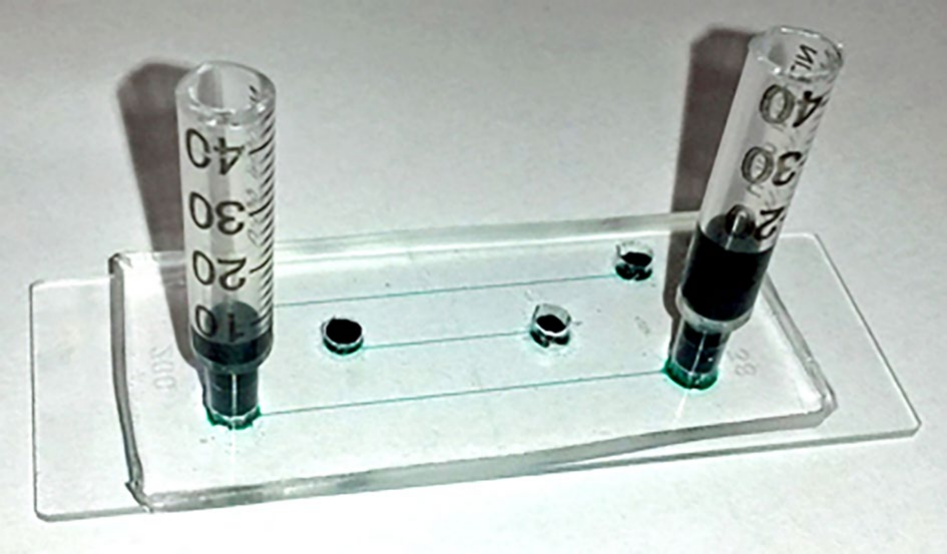
The researchers applied hydrostatic pressure to create a fluid flow inside themicrochannel where sperm rheotaxis was studied. Microchannels with dimensions of 200 μm × 20 μm (W × H) and a length of 3.6 μm were employed. Themicrochannel in between reservoirs with syringes mounted at ends were used. To make the channel more visible, food colours were employed.

**Figure 2:**
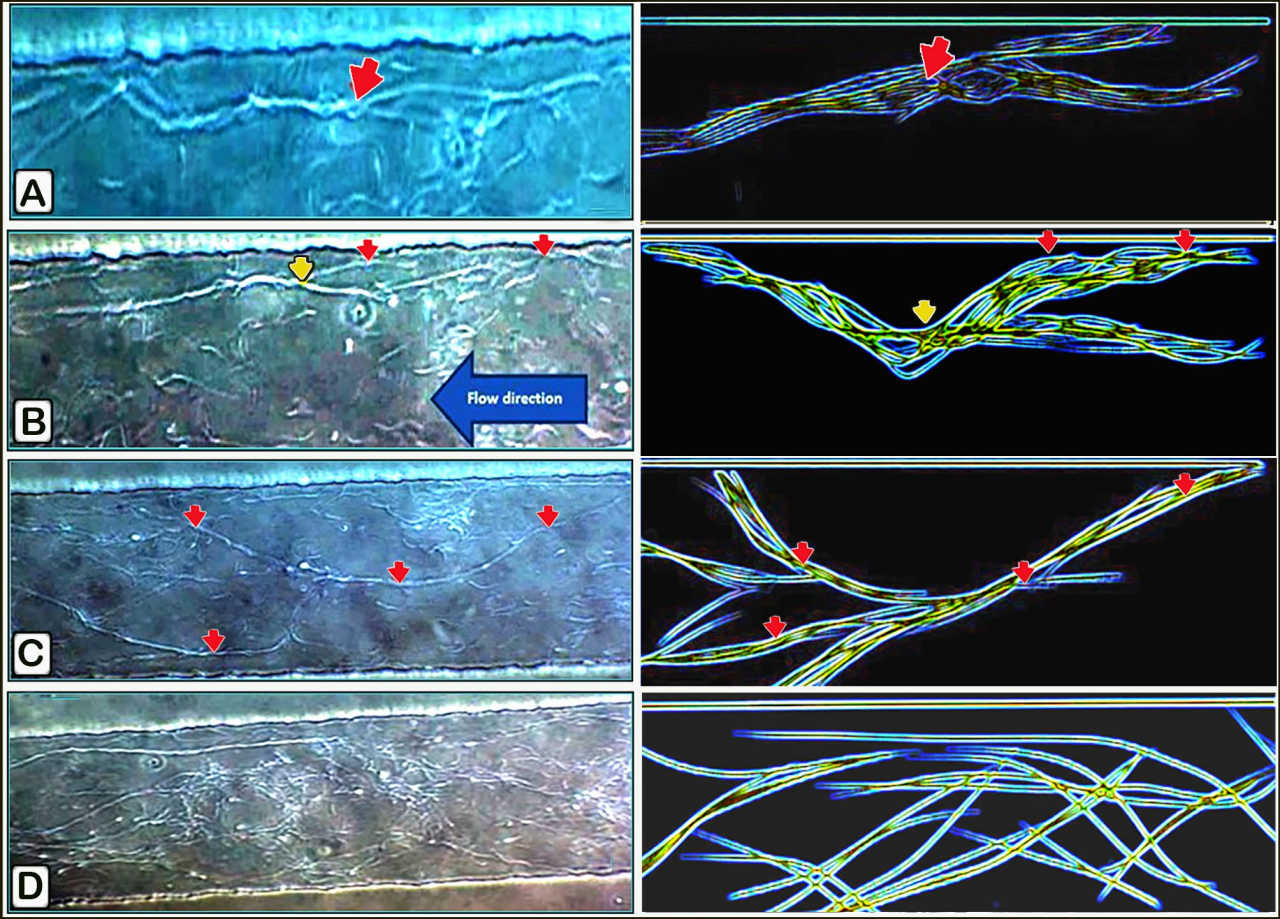
Bundle interconnections and attachments to the walls. Video taken from phase contrast microscope. Images from phase-contrast microscopy and drawings accompany each image for presentation. (**A**) The connection between two threads by spiral movement to resist the flow (red arrow). (**B**) Attachment between the bundles and wall of the channel (red arrow); at the same time, they are connected to two other bundles (yellow arrow). (**C**) Sperm bundles inside the microfluidic channel start to be connected (red arrows) with each other, making a net of sperm bundles. (**D**) The formation of a network of sperm bundles.

The sperm bundles moved parallel to each other when present in a slow fluid flow; however, they start to overlap and stick to any stationary object so as not to be swept away with the flowing current when the velocity of the flow is increased. The formation of the bundle begins with few sperm getting close to each other, and they begin to move synchronously and wrap around each other and then stick to the adhesive substance. Figures 1 and 2 show sperm approaching one another forming a connecting point because of the tails wrapping around each other.

### Adhesion of the bundles (Videos 5, 6, 7)

When a drop of diluted semen was loaded on the microfluidic device and a flow was generated, sperm bundles were noticed to move against the direction of the flow. The bundles approach the walls of themicrochannels, and the free heads in the initial part of the bundle adhered tightly to them (Video 5). They also stick to any stationary particles such as debris in their path as an attempt to resist drifting with the current. With time, these bundles are long threads that catch other single sperm and shorter bundles (Video 6). When the flow started to get slower, the long threads of sperm started to make a net of sperm threads (Video 7; Fig. 2).

### Response to velocity (Video 8)

At high flow velocity (V > 33 µm/s), the spiral movements of threads are increased as an attempt to catch many individual sperm forming bundles better resist the drifting force of the flow. They also attempt to attach to the sidewall of themicrochannel.

### Descriptions of sperm bundles in sharakasi chickens using various microscopy methods

Sperm bundles were identified using light microscopy (LM) as an accumulation of sperm heads and coiled tails. Sperm bundles of differing aggregations were also identified, as twisted heads and aggregates of the flagellum, as adhered tails of multiple sperm, as having the head of the sperm attached to the tail, as having the head of the sperm with a bent nucleus, as multiple adhered tails of sperm, and as sperm heads adhered together by an agglutination substance using transmission electron microscopy (TEM). Scanning electron microscopy (SEM) revealed a sperm bundle as an aggregate of sperm heads covered by a coat, with the sperm aggregations displaying a network of attached wrapped tails.

### The use of histological tools to investigate the formation of sperm bundles

The morphology and ultrastructure of the sperm, as well as the formation of bundles, were investigated using a light microscope (semi-thin section), scanning electron microscopy (SEM), and transmission electron microscopy (TEM), and semen smears were stained with acridine orange and examined using epifluorescence microscopy.

**Figure 3:**
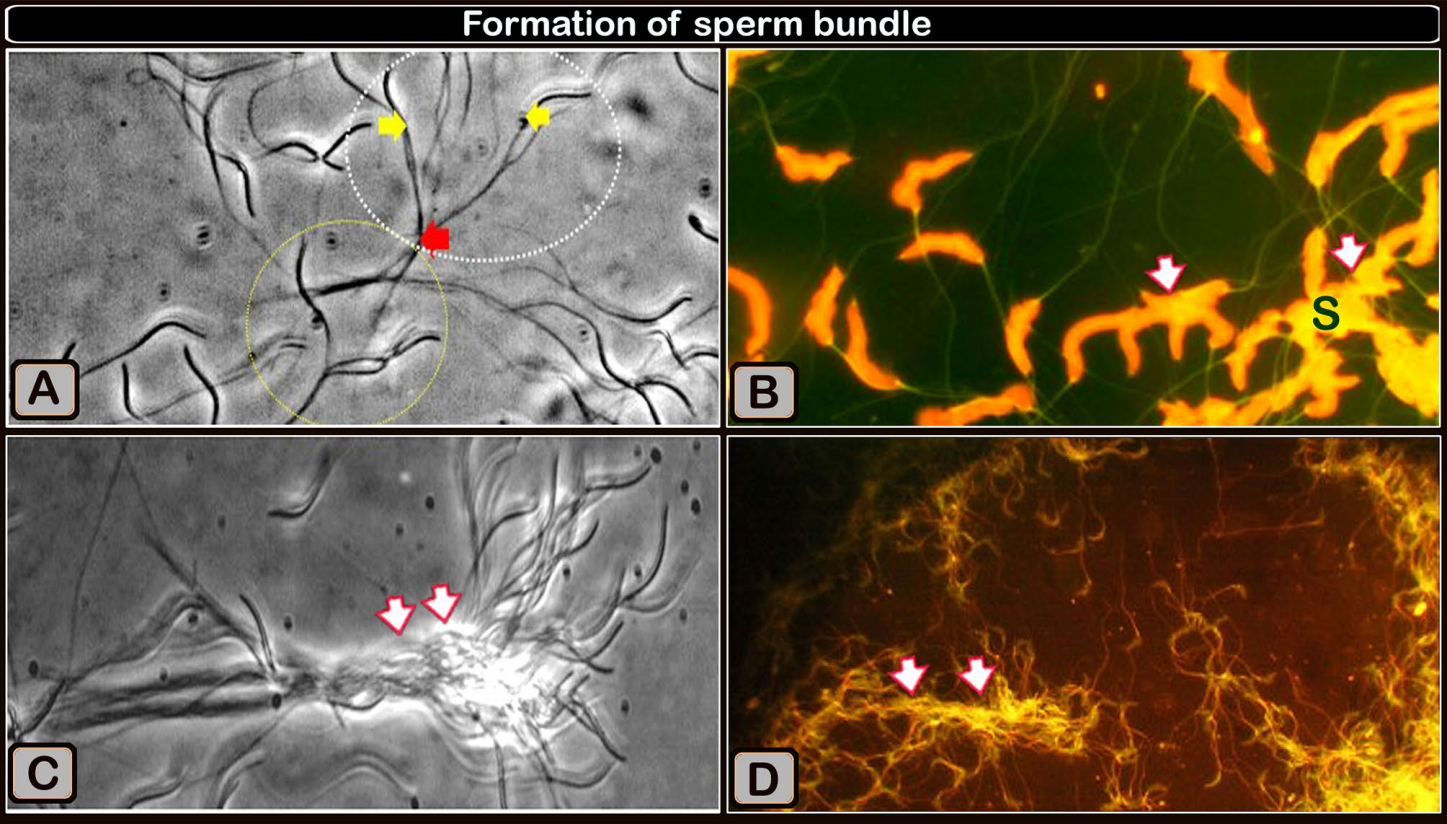
Formation of sperm bundles using a phase contrast microscope and an acridine orange-stained sperm smear showing that the sperm head sticking together. (**A**) The early formation of sperm bundles started with sperm (white circle) and three sperm (yellow circle), and the spiral started at the tail and finally at the head. (**B**) Micrograph of a semen smear stained with acridine orange showing that the sperm head stucking together (arrows). The secretion covers the heads (S). Magnification ×1000. (**C**) The developed of large bundles transported through the flow in the micro fluid channel (using a high-speed camera at 950 frames/s). (**D**) Acridine orange-stained sperm smear micrograph showing the formation of large bundles (arrows). Magnification: ×200.

**Figure 4:**
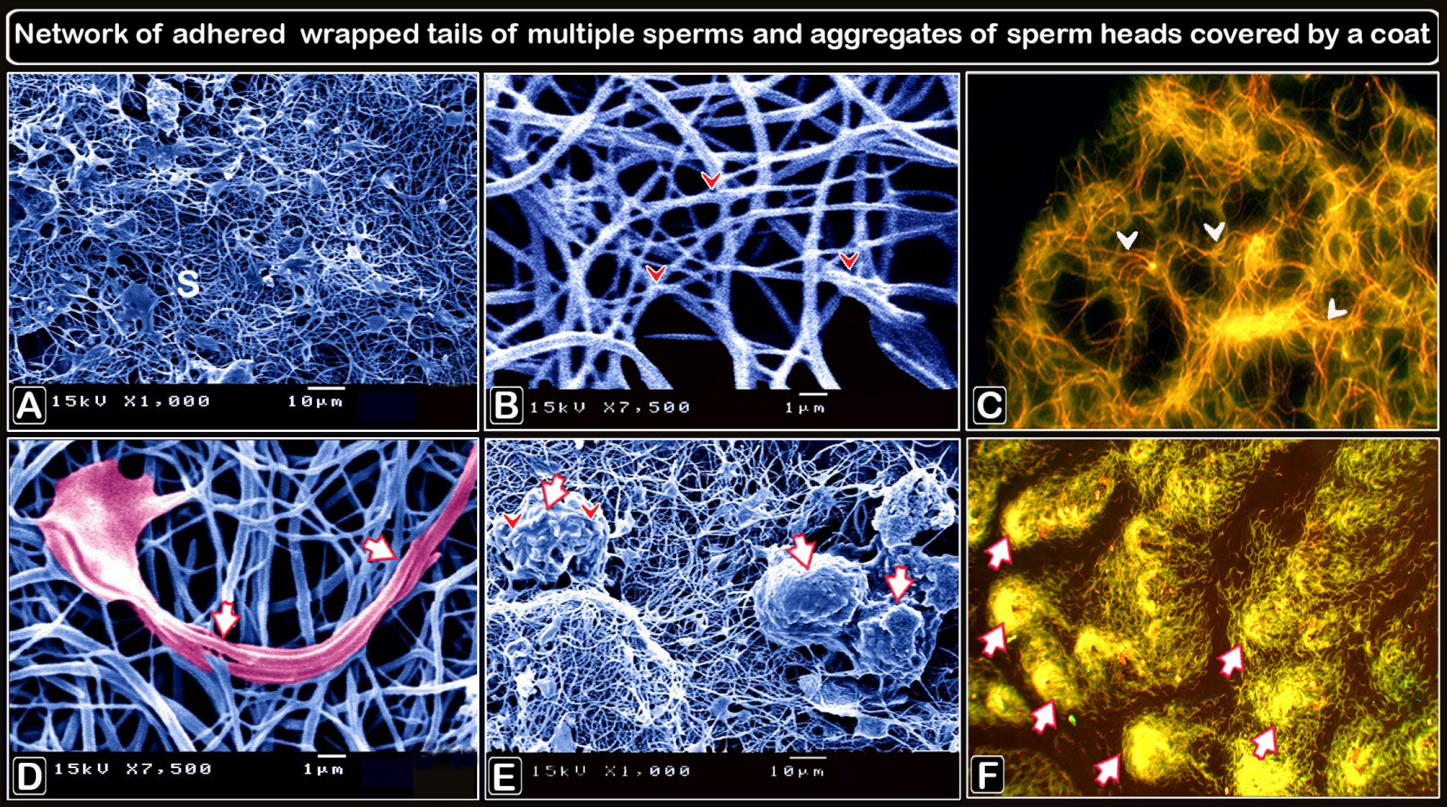
Scanning electron micrograph images of sperm bundles and an acridine orange-stained sperm smear. (**A**,**B**,**D**,**E**) are digitally colored scanning electron micrographs of sperm, whereas C and F are micrographs of sperm smears stained with acridine orange and showing a network of adherent wrapped tails of multiple sperm. (**A**–**C**) Aggregations of sperm (s) shown as a network of adhered tails (arrowheads). (**D**) Multiple sperm of adhered wrapped tails (with the agglutinating substance, pink outlined, arrows). (**E** and **F**) Sperm head aggregates (arrows) coated by a agglutinating material (arrowheads). The sperm formed bundles with few twister-like structures (**F**). Magnification of (**C**) ×400 and (**F**) ×200.

The sperm heads adhered together, and covered with secretory material, as shown by the acridine orange staining of the sperm smears (Fig. 3B) and this resulted in the formation of large bundles (Fig. 3D). The sperm bundles consisted of aggregations of sperm that exhibited a network of adhered tails (Fig. 4A–C). The sperm bundle consisted of adhered wrapped tails of multiple sperm (Fig. 4D). A secretion (Fig. 4E,F) covers the sperm heads of the bundle.

**Figure 5:**
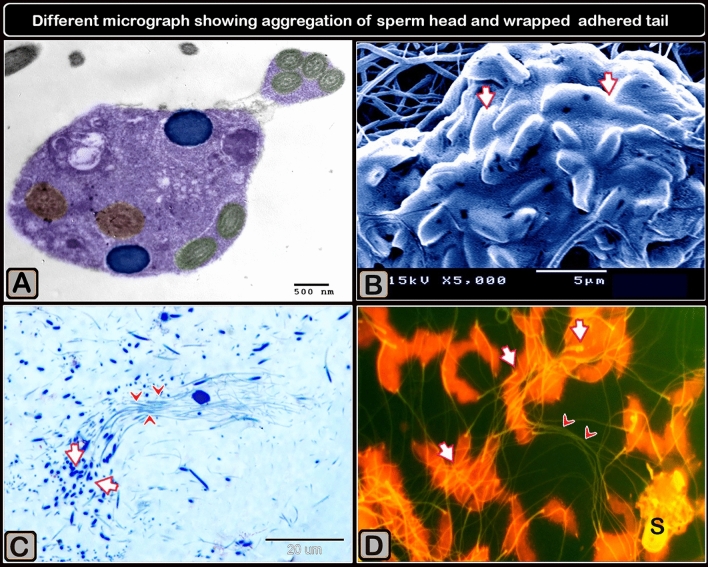
Digitally colored transmission electron micrographs demonstrating connected tails in a sperm bundle and agglutination material connecting sperm heads. (**A**) Adhered sperm tails from numerous sperms. Note of how the tail appears in longitudinal (arrows) and transverse (arrowheads) views. (**B**) The sperm's head (arrowhead) is linked to the tail (arrow). (**C**) Several sperm tails attached (arrowheads). (**D**) Agglutination material (AS, blue color) connects four sperm heads (violet color).

**Figure 6:**
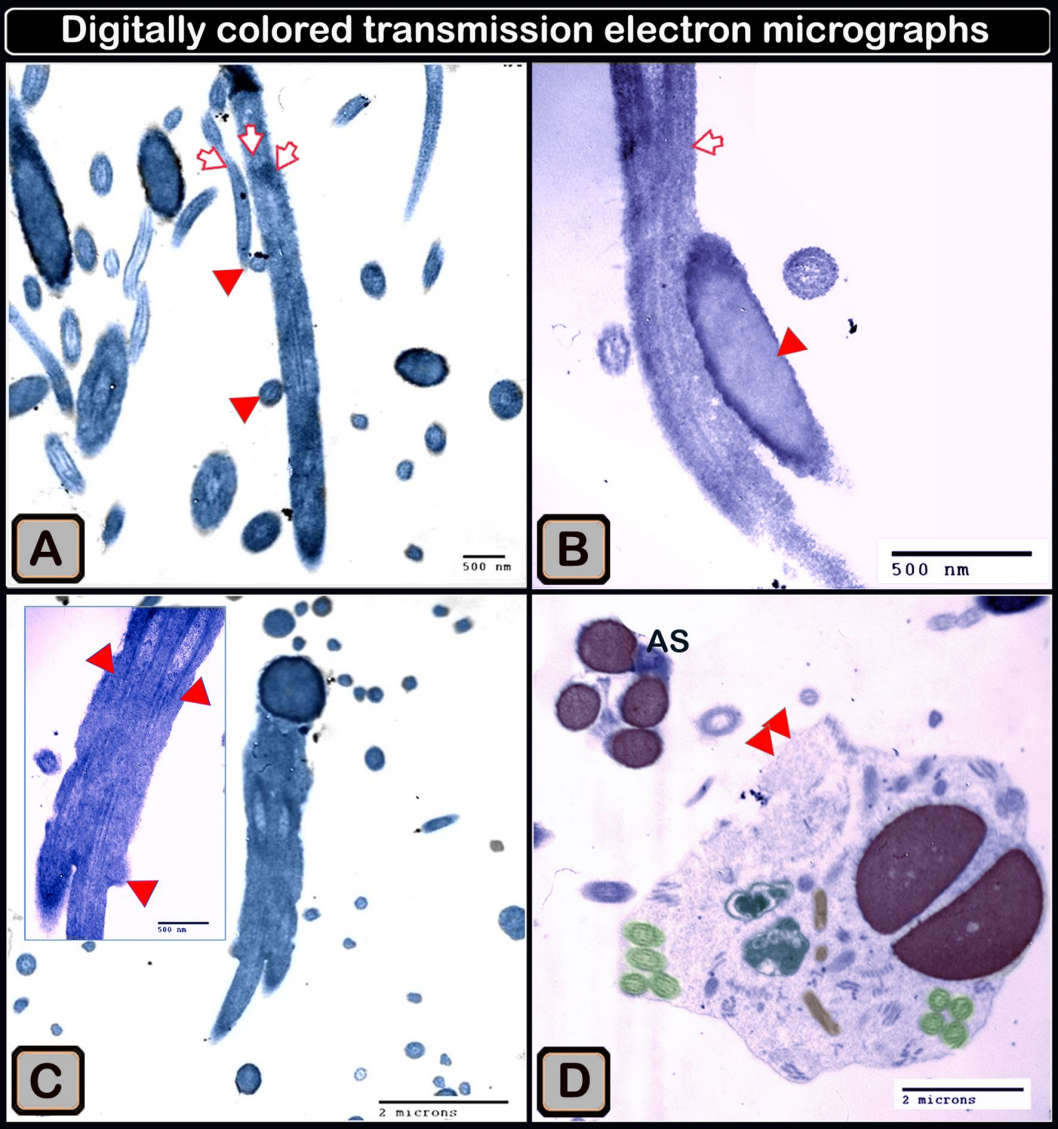
Various micrographs displaying sperm head and wrapped tail aggregation using various methods. (**A**) Digitally colored transmission electron micrograph of a sperm bundle in a cut section show twisted sperm heads having two portions of the nucleus (blue color) and several potions of the flagellum (green color). (**B**) Digitally colored scanning electron micrograph showing a cluster of sperm medusa-like heads that appeared to be coated (arrows). (**C**) Semithin sections showing sperm head aggregation (arrows) and wrapped tails (arrowheads). (**D**) Acridine orange-stained sperm smear micrograph shows aggregates of sperm heads (arrows) and wrapped adherent tails (arrowheads). Note that the adhesive substance (S) covered the sperm head. Magnification of (**D**) ×1000.

Using transmission electron microscopy, we showed that the sperm bundles had adhered tails (Fig. 6A,C), with the heads adhered to the tail (Fig. 6B), or the heads being attached to the tails (Fig. 6D). The head of the sperm in the bundle was bent, presenting two sections of the nucleus in the cut section (Fig. 6D). In the bundle in the cut section, sperm had twisted heads with two sections of the nucleus and multiple portions of the flagellum (Fig. 5A).

Scanning electron microscopy was used to detect the sperm heads in the bundle covered by a secretion or coat (Fig. 6B), which revealed that the sperm bundles were held by extracellular substances. The agglutinating substance had been concentrated at the sperm heads (medusa head-like assembly; Fig. 5B) and extended distally, with a shiny yellow appearance under a fluorescence microscope when dyed with acridine orange (Fig. 6C). This substance is clearly visible using scanning microscopy and is considered the agglutinating matter. Semi-thin sections (Fig. 5C) and acridine orange-stained semen smears revealed that the sperm bundle contained tightly packed heads and coiled tails (Fig. 5D).

**Figure 7:**
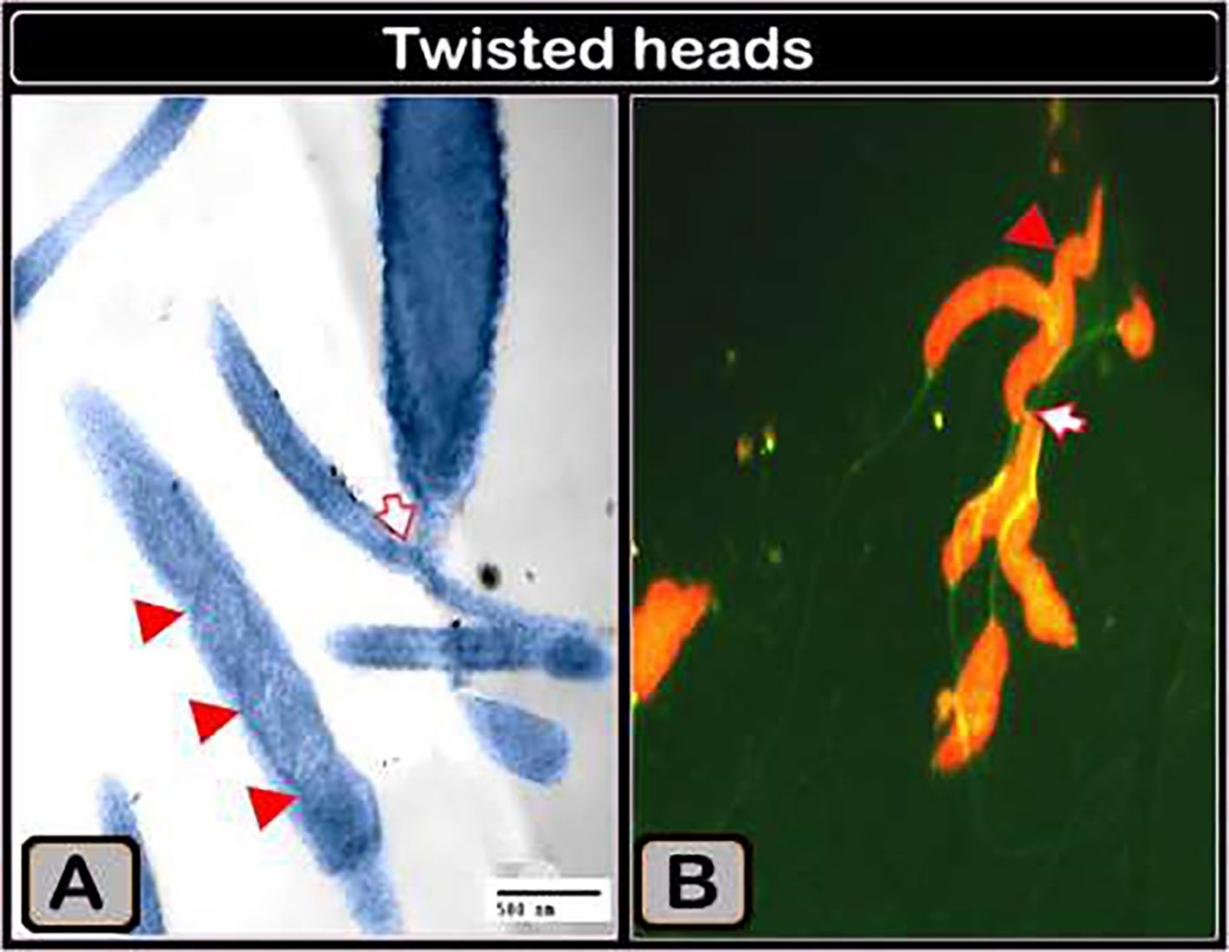
(**A**) Digital colored transmission electron micrograph and (**B**) acridine orange-stained semen smear showing twisted heads and attachment of sperm heads and tails (arrow). Magnification of (**B**) ×1000.

Using a transmission electron microscope (Fig. 7A), it was also noted that the sperm head was twisted and that the nucleus resembled a helical shape, which was supported by semen smears stained with acridine orange and studied with a fluorescence microscope (Fig. 7B).

## Discussion

The finding that Sharkasi spermatozoa agglutinate, forming motile thread-like bundles, is interesting. The properties of these bundles provide insight into the role they may play in sperm uptake and storage in the SSTs.

After mating, sperm are deposited into the vagina and undergo an intense selection process resulting in only a limited number of sperm entering the SSTs^15,16^. To date, the mechanism by which sperm enter and evacuate the SSTs is unknown. In domestic fowl, spermatozoa are maintained inside the SSTs for extended durations ranging from 2 to 10 weeks depending on the species^6^. The controversy remains with regard to the state of the sperm during storage inside the SSTs; are they in a state of movement or inactivity? In other words, how do sperm keep their position inside the SSTs for prolonged periods?

Forman^4^ proposed that sperm residency and egress from the SST can be explained on the basis of sperm cell motility. The author suggested that sperm maintain their position by swimming against a fluid current generated by SST epithelial cells and that sperm egress from the SST when their velocity falls below a point at which retreated movement begins due to a lack of energy. Zaniboni^5^ confirmed the presence of aquaporin’s 2, 3, and 9 within the apical portion of the SSTs epithelial cells which may indirectly support Forman’s model of sperm storage. In the current study, we found that nearly half of Sharkasi sperm show positive rheotaxis in the presence of a flowing fluid and that agglutinated sperm bundles increase the number of sperm exhibiting positive rheotaxis, although the agglutination slows down their velocity. How sperm ascend to the oviduct in birds to reach the fertilization site is not fully understood. In mammals, follicular fluid chemoattracts spermatozoa^7^. However, it is thought that chemo-attractants guide the sperm at an approaching distance^7^. Therefore, other mechanisms are responsible for sperm transportation. The ability of sperm to orient itself and swim against the flow of oviductal fluid secreted post copulation was reported to be a major factor responsible for sperm guidance in mice^7^. Parker^17^ assumed that sperm traverse the oviduct by swimming against the ciliary current in birds and reptiles. Although it was not proven experimentally in vivo for birds, Adolphi^18^ was the first to observe that avian sperm exhibit positive rheotaxis when a slow current is generated in a thin layer of fluid contained between a coverslip and a glass slide using a strip of filter paper. Hino and Yanagimachi^19^ installed mouse ovaries-oviduct-uterus complex in a perfusion collar and injected 1 µl of India ink in the isthmus to visualize the fluid flow inside the oviduct. They noted a very active contraction and relaxation movement in the oviduct in which all boluses of ink moved steadily toward the oviduct’s ampulla. The authors highlighted the importance of the flow of oviduct fluid from the lower to upper parts of the oviduct for sperm ascension and fertilization. In chickens and turkeys, Brillard^20^ reported that the migration of spermatozoa, from the entrance to the vagina where they are deposited to the uterovaginal junction where they are stored, is achieved through their active motility. However, this motility is not needed between the uterovaginal junction and the infundibulum because sperm are transported by passive displacement. With knowledge of these previous suggestions and the results obtained in the current study it can be assumed that the ability of sperm to move against the flow (rheotaxis) is one of the attributes on the basis of which the selection process takes place. And this determines the passage of sperm through the vagina and their arrival in the SSTs for storage. It may also contribute to the process of sperm penetration into the SSTs and their residence for a certain period and then their exit when their velocity begins to slow down, as Forman^4^ hypothesized.

On the other hand, Matsuzaki and Sasanami^21^ suggested that avian sperm undergo motility changes from quiescent to motile in both male and female reproductive tracts. Suppression of resident sperm motility within SSTs was proposed to explain prolonged sperm storage, which is followed by motility restoration after release from the SSTs^21^. Under hypoxic conditions, Matsuzaki et al.^1^ reported high lactic acid production and release in the SSTs, which may lead to suppression of resident sperm motility. In this case, the significance of sperm rheotaxis is manifested during sperm selection and uptake but not during storage.

The sperm agglutination mode was suggested to be a plausible elucidation for the prolonged storage period of sperm within the SSTs because this is the pattern of sperm residency common among domestic birds^2,22,23^. Bakst et al.^2^ observed that most sperm adhere to each other, forming bundle-like agglutinations, and lonesome sperm are rarely seen in the SSTs of quails. On the other hand, Wen et al.^24^ observed more scattered sperm and fewer sperm bundles in the lumen of SSTs in chickens. From these observations, it can be assumed that the tendency of sperm to agglutinate differs among avian species and between sperm in the same ejaculate. In addition, Van Krey et al.^9^ suggested that the random dissociation of agglutinated sperm is responsible for the gradual exit of spermatozoa into the lumen of the oviduct. According to this assumption, sperm with less capacity to agglutinate should egress from SSTs first. In this case, the sperm capacity to agglutinate could be a factor influencing the outcomes of sperm competition in promiscuous birds. In addition, the longer the period the agglutinated sperm take to dissociate, the longer the duration of fertility will be.

Even though aggregations of sperm and their adhesion in bundles have been observed in several studies^2,22,24^, they have not yet been described in detail because of the difficulty of observing them kinematically inside the SSTs. Few attempts have been made to study sperm agglutinations in vitro. Extensive but transient aggregations were observed when a delicate wire was withdrawn through a hanging drop of semen. This caused an elongate vesicle to project from the drop, simulating a sperm gland. Due to three-dimensional limitations and the short time the drop takes to dry, the whole unit deteriorated quickly^9^. In the current study using Sharkasi chickens and a microfluidic chip, we were able to describe how these bundles are formed and how they move in detail. The sperm bundles are formed as soon as semen was collected and were found to swim in a spiral-like movement and show positive rheotaxis when present in a flow. In addition, it was observed that sperm bundles increase the linearity of motility compared to lonesome sperm when observed macroscopically. This indicates that sperm agglutination can occur before SST penetration and that their formation is not subject to being confined in a small area due to pressure as suggested before (Tingari and Lake^12^). During sperm bundle formation, sperm swim synchronously until they form connection sites, then their tails are wrapped around one another and sperm heads remain free, but the tails and distal sperm are adhered together by an adhesive substance. Therefore, the free heads of the bundle are responsible for the movement by dragging the rest of the bundle. Scanning electron microscopy of sperm bundles revealed adhered sperm heads covered with a copious adhesive substance, indicating that sperm heads attach in stationary bundles that may occur after reaching the storage site (SSTs).

When semen smears are dyed with acridine orange, the extracellular adhesive substance surrounding the sperm is visible under a fluorescence microscope. This substance allows the sperm bundles to stick to any surrounding surface or particles and to cling to them so that they do not drift with the surrounding currents. Therefore, our observations shed light on the role that sperm adhesion in the form of motile bundles. Their ability to swim against the flow, as well as their ability to stick to adjacent surfaces maintain sperm residence for a extended time inside SSTs.

Rothschild^25^ used a hemocytometer chamber to examine the swimming distribution of bull spermatozoa in a drop of suspension by taking micrographs through the chamber when the optical axis of the microscope was vertical and horizontal. The results showed that sperm are attracted to the surfaces of the chamber. The authors suggested that there might be hydrodynamic interactions between the sperm and the surfaces. Taking this into account, as well as the ability of Sharkasi chicken sperm to form sticky bundles, may increase the possibility of sperm adhering to the SST walls and being stored for extended time.

Baccetti and Afzeliu^26^ reported that the spermatozoal glycocalyx is essential for gamete recognition and agglutination. Forman^10^ observed that hydrolyzing α-glycosidic bonds in the glycoprotein-glycolipid coating by treating fowl spermatozoa with neuraminidase resulted in decreased fertility without affecting sperm vitality. The authors speculated that manipulation of the glycocalyx by neuraminidase perturbed sperm sequestration in the utero-vaginal junction, which consequently decreased fertility. Their observations could not discount the possibility that neuraminidase treatment may have reduced sperm oocyte recognition. Forman and Engel^10^ found that fertility rates were decreased when hens were intravaginally inseminated with neuraminidase-treated spermatozoa. However, intramagnal insemination with neuraminidase-treated spermatozoa had no effect on fertility compared with control birds. The authors concluded that alterations in the glycoprotein-glycolipid cover surrounding the sperm membrane reduce the fertilizing ability of the sperm by perturbing sperm sequestration in the utero-vaginal junction, which in turn increases the rate at which sperm are lost from the uterovaginal junction but does not affect sperm-oocyte recognition.

In turkeys, Bakst and Bauchan^11^ found small vesicles and membrane fragments in the lumen of SSTs and observed that some of these particles were fused with the sperm membrane. The authors speculated that these interconnections might contribute to prolonged sperm storage in SSTs. However, the researchers did not clarify the source of these particles, whether they are secreted from the epithelial cells of SSTs, produced and secreted from the male reproductive system, or from the sperm themselves. Also, whether these particles are responsible for agglutination. Grützner et al.^27^ reported that the epithelial cells of the epididymis produce and secrete a specific protein(s) that is needed for the formation of sperm bundles in monotremes. The authors also reported that the dispersal of these bundles is dependent upon epididymal protein(s) interactions. Nixon et al.^28^ found that the epididymal secreted protein, acidic cysteine-rich osteonectin; SPARC, is involved in sperm bundle formation in both short-beaked echidna and platypus. With the dispersal of these bundles being associated with the loss of this protein.

In the current study, ultrastructure analysis using electron microscopy revealed that sperm adhered to large amounts of dense substances. These substances are thought to be responsible for agglutination, and it is condensed in-between and around the adhered heads but found at a lower concentration in the tail region. We suggest that this agglutinating substance is secreted from the male reproductive system (epididymis or vas deferens) with the semen because we frequently observed that sperm are sequestered from the lymphatic fluid and seminal plasma at the time of ejaculation. It was reported that when fowl sperm pass through the epididymis and vas deferens, they undergo maturational changes supporting their ability to bind proteins and in acquiring plasma lemma-associated glycoproteins^29,30^. These proteins persist on the membranes of resident sperm in the SSTs suggesting that these proteins may influence the acquisition of sperm membrane stability^30^ and determine their fertilizing capacity^31^. Ahammad et al.^32^ reported that sperm obtained from different parts of male reproductive system (from testes to the distal vas deferens) displayed gradually increasing survival rates under liquid storage conditions, irrespective of the storage temperature, and also increasing capacity to survive in the hen oviduct after artificial insemination.

Sharkasi chicken sperm bundles have different features and functions compared to those discovered in other species, such as echidna, platypus, wood mouse, deer mouse, and guinea pig. In Sharkasi chickens, the formation of sperm bundles decreases its swimming velocity compared to individual sperm. However, these bundles increase the percentage of sperm showing positive rheotaxis and enhance the sperm's ability to stabilize themselves in a dynamic environment. Therefore, our findings strengthen the previous suggestions that sperm agglutination in SSTs is associated with a prolonged duration of sperm storage. We also speculate that the tendency of sperm to form bundles may control the rate of sperm loss from SSTs, which, as a result, may change the outcomes of sperm competition. According to this speculation, sperm with low agglutination capacity evacuate SSTs first, while males possessing sperm with high agglutination capacity generate most of the progeny. The formation of sperm bundles in monotremes is advantageous and affects the paternity proportions but using a different mechanism. In echidna and platypus, sperm are arranged parallel to each other to increase the bundle’s forward velocity^28^. The motility of the bundle in echidna is approximately three times faster than that of lonesome sperm^27^. The formation of such sperm bundles in echidna is considered an evolutionary adaptation to assert dominance because the females are promiscuous and commonly mate with several males. Consequently, spermatozoa from different ejaculates are in high competition to fertilize the ova^33^.

The agglutinated sperm bundles in Sharkasi chickens are easily observed using a phase-contrast microscope, which is considered advantageous because it allows easy study of sperm behavior in vitro. The mechanism by which the formation of sperm bundles benefits reproduction in Sharkasi chickens also differs from that seen in some eutherian mammals representing cooperative sperm behavior, such as wood mice, where some spermatozoa show altruistic behavior by helping other related individuals reach the ovum and compromise their own fertilizing ability^34^. Another example of sperm cooperative behavior is discovered in deer mice, where sperm are able to recognize and aggregate with the most genetically related sperm and form cooperative groups to increase their velocity against unrelated sperm^35^.

The results obtained in this study do not contradict Foman's theory to explain the long-term storage of sperm within SSTs. The researcher reported that sperm spend long periods of time in constant motion against a flowing stream of epithelial cells lining the SSTs and that after a while the sperm's energy reservoir depletes and, as a result, the velocity decreases, allowing the drainage of low-energy sperm with the flowing fluid from the lumen of the SSTs out into the oviduct lumen. We observed in the current study that half of lonesome sperm display the ability to swim against flowing fluid and that their adhesion in bundles increases their ability to show positive rheotaxis. In addition, our findings do not contradict those of Matsuzaki et al.^1^ who reported increased lactic acid secretion in the SSTs that may suppress resident sperm motility. However, our results describe the formation of the motile sperm bundles and their rheotactic behavior when present in a dynamic environment inside micro-channels as an attempt to elucidate their behavior in the SSTs. Future research will probably focus on identifying the chemical composition and source of the agglutinating material, which will undoubtedly aid researchers in devising new methods for storing liquid sperm and increasing fertility duration.

## Materials and methods

### Birds

Fifteen thirty-week-old naked neck Sharkasi roosters (homozygous dominant; Na Na) were selected and used as sperm donors in the study. Birds were raised at the Research Poultry Farm, Faculty of Agriculture, Assiut University, Assiut Governorate, Egypt. Birds were housed in individual cages (30 × 40 × 40 cm), exposed to a lighting program (16 h light: 8 h dark), and fed a commercial diet containing 160 g crude protein, 2800 kcal metabolizable energy, 35 g calcium, and 5 g available phosphorus per kg diet.

### Semen collection and preparation

The abdominal massage method was used to collect semen from roosters according to^36,37^. In total, 45 semen samples were collected over a period of 3 days from the 15 males. Ejaculates (n = 15/day) were immediately diluted with Beltsville poultry semen extender 1:1 (v:v) comprising potassium diphosphate (1.27 g), sodium glutamate monohydrate (0.867 g), fructose (0.5 g), sodium acetate anhydrous (0.43 g), Tris (hydroxymethyl)-aminomethane (0.195 g), potassium citrate monohydrate (0.064 g), potassium monophosphate (0.065 g), magnesium chloride (0.034 g), and H_2_O (100 ml); pH = 7.5, and osmolality 333 mOsm/kg^38^. Diluted semen samples were initially examined under a light microscope to ensure good quality of sperm (vigorous motility) and then kept in a water bath at 37 °C until use within half an hour of collection.

### Evaluation of sperm velocity and rheotaxis evaluation

The sperm kinematics and rheotaxis were described using the microfluidic device system. Semen samples were further diluted to 1:40 in Beltsville poultry semen extender and were loaded into the microfluidic device (see below), and the kinetic parameters were determined through a computer-assisted sperm analysis (CASA) system^39^ that was previously developed for the characterization of sperm motion in microfluidic environments (Department of Mechanical Engineering, Faculty of Engineering, Assiut University, Egypt). The plugin can be downloaded from URL: http://www.assiutmicrofluidics.com/research/casa^39^. Curvilinear velocity (VCL, µm/s), straight-line velocity (VSL, µm/s), and average path velocity (VAP, µm/s) were measured. Videos of sperm cells were taken with an Optika XDS-3 inverted microscope with phase contrast (at 40× objectives) coupled to a Tucson ISH1000 camera at 30 frames/s for 3 s. A minimum of three fields and 500 sperm tracks were examined per sample using the CASA software. Recorded videos were processed using a home-developed CASA. The definition of motility in the CASA plugin is based on sperm swimming speed versus flow speed without including other parameters, such as side-to-side movement, as this was found to be more reliable in fluid flow. The rheotactic movement was described as sperm swimming against the direction of fluid flow. Sperm exhibiting rheotaxis were divided by the number of motile sperm; with static sperm and those swept out by convective flow, were removed from the count.

### Microfluidic device fabrication

All chemicals used were obtained from Elgomhoria Pharmaceuticals (Cairo, Egypt) unless otherwise specified. The device was fabricated as described by El-sherry et al.^40^, with some modifications. Materials for microchannel fabrication included glass wafers (Howard Glass, Worcester, MA), SU-8-25 negative resist (MicroChem, Newton, CA), diacetone alcohol (Sigma Aldrich, Steinheim, Germany), and polydimethylsiloxane PDMS (Syllgard-184, Dow Corning, Midland, MI). Microchannels were fabricated using soft lithography^41^. First, a transparency mask bearing the required microchannel design was printed using a high-resolution printer (Prismatic, Cairo, Egypt and Pacific Arts and Design, Markham, ON). Masters were prepared using glass wafers as a substrate. Wafers were cleaned in acetone, isopropyl alcohol, and deionized water and then coated with a 20 µm thick layer of SU8-25 by spin coating (3000 rpm, 1 min). The SU-8 layer was then soft-baked (65 °C, 2 min, and 95 °C, 10 min) and exposed to UV light for 50 s. A postexposure bake was performed at 65 °C for 1 min and 95 °C for 4 min to cross link the exposed SU-8 layer, which was then developed in diacetone alcohol for 6.5 min. The wafers were hard baked (200 °C for 15 min) to further harden the SU-8 layer.

PDMS was prepared by mixing the monomer and curing agent at a ratio of 10:1 by weight and then degassed in a vacuum desiccator and poured on to the SU-8 master. PDMS was cured in an oven (120 °C, 30 min), and channels were then cut, peeled off the master, and punched to allow for tubing connections at the inlet and outlets of microchannels. Finally, a portable corona treater (Electro-Technic Products, Chicago, IL) was used to permanently bond the PDMS microchannels to a microscope glass slide as described elsewhere^42^. The dimensions of the microchannels used in the present study were 200 µm × 20 µm (W × H) and 3.6 cm long.

### Flow generation

Hydrostatic pressure-induced liquid flow inside the microchannel was achieved by keeping the liquid level at the inlet reservoir higher than at the output reservoir with a height difference Δh^39^ (Fig. 1).

Average velocity inside the microchannel can then be calculated using the Darcy-Weisbach equation,

$$\Delta h = fLDhVav2g$$

where *f* is the friction factor defined as *f* = *C/Re* for laminar flow in a rectangular duct, where *C* is a constant dependent on channel aspect ratio, *L* is the microchannel length, *V*_*av*_ is the average velocity inside the microchannel, *D*_*h*_ is the channel hydraulic diameter, and *g* is the gravitational acceleration. Using this equation, average velocity inside the channel can be calculated using the following equation:

$$V_{av} = \frac{{2\rho gD_{h} \Delta h}}{C\mu L}$$

A velocity profile forms throughout a channel, and was calculated using the following equation for channels with an aspect ratio of less than 0.5.

1 $$\frac{V}{{V_{av} }} = \left( {\frac{m + 1}{m}} \right)\left( {\frac{n + 1}{n}} \right)\left[ {1 - \left( \frac{y}{b} \right)^{n} } \right]\left[ {1 - \left( \frac{z}{a} \right)^{m} } \right]$$

where V is liquid velocity at any location in the channel, *V*_*av*_ is average liquid velocity, *a* is the channel width, *b* is the channel height, *y* and *z* are the two coordinates measured from channel centerline along channel height and width respectively, and *m* and *n* are two numerical factors calculated according to

2 $$m = 1.7 + 0.5\;\alpha^{ - 1.4}$$

3 $$\begin{array}{*{20}l} {n = 2} \\ {n = 2 + 0.3\,(\alpha - 1/3)} \\ \end{array} \;\;\;\left\{ {\begin{array}{*{20}c} {\alpha < \frac{1}{3}} \\ {\alpha \ge \frac{1}{3}} \\ \end{array} } \right.$$

Hydrostatic flow generation is a simple and low-cost method to generate flow inside microchannels and does not suffer from pulsating flow that is typical of syringe pumps^43^. The average velocity inside the microchannel can be calculated using the Darcy-Weisbach equation^44^. The velocity profile inside the channel was calculated for channels with an aspect ratio of less than 0.5^45^. The average liquid velocity was kept at 33 ± 5 µm/s.

### Histological investigation of semen samples

#### Fixation of semen samples

Following semen collection and dilution, samples (n = 6) were preserved in Karnovsky fixative^46^ (Table 1) and two aliquots were made for each sample for scanning electron microscopy analysis and resin fabrication of semithin and ultrathin sections. Other tubes holding unfixed samples were utilized to prepare smears for staining with acridine orange stain. Semen smears were stained by Acridine orange stain, a cationic dye that stains protein-containing membranous vesicles such as secretory vesicles, membrane-bound acidic compartments, and acidic lysosomes. Acridine orange undergoes a metachromatic reaction that is connected to the release of green and red fluorescence. Acridine orange reacts with membrane-bound vesicles, causing them to stain orange or red. Acridine orange is used to detect secretory vesicles and lysosomes^47–50^.

**Table 1:** Components of the Karnovsky fixative sand phosphate buffer.

Fixative	Components	Amount
Karnovsky fixative	Paraformaldehyde, 25% freshly prepared	10 ml
	Glutaraldehyde 50%	10 ml
	Na-phosphate buffer (0.1 M, pH 7.4)	50 ml
	Distilled water	30 ml
N a-phosphate buffer (0.1 M, pH 7.4)	**Solution A**	
	Na_2_HPO_4_·2H_2_O	17.02 g
	Distilled water	600 ml
	**Solution B**	
	NaH_2_PO_4_·H_2_	6 g
	Distilled water	200 ml
	**Using solution**	
	Solution A	580 ml
	Solution B	219 ml

#### Preparation of acridine orange acid stain

Components of *stain* Distilled water, acridine orange, glacial acetic acid.

*Reagent preparation* A stock solution of 50 mg acridine orange was prepared in 10 ml of distilled water and stored in the refrigerator. To make a working solution, it was combined with 1 mL of acridine orange stock solution and 0.5 mL of glacial acetic acid in 50 mL of distilled water.

*Staining method* A smear in made on a clean slide, and then air-dry. After that, the slide was fixed with methanol and air dried again. It is then placed in a trough containing an acridine orange staining working solution (i.e., 0.01 percent) for 20 min. The slides were gently washed and dried before being analyzed using a microscope (model Letiz DM 2500) with external fluorescent units (Leica EL 6000).

#### Resin embedding section for semi thin section

1. After two drops of diluted semen were fixed in Karnovsky fixative for 2 h at 4 °C and then processed as directed by^51^. Fixation applied an equivalent volume of Karnovsky fixative to the diluted semen for 2 h at 4 °C.
2. The fixed samples were centrifuged, and the pellet was suspended in fresh PBS buffer. Washing three times with phosphate buffer solution pH 7-2-7-4 (Table 1) and centrifuged after each wash
3. For two hours at room temperature, samples were centrifuged and post fixed in 10% osmium tetroxide made up in 0–1 M phosphate buffer pH 7-2-7-4 and 2–5 mM calcium chloride.
4. The samples were centrifuged, and then the pellet was suspended and washed in 50% ethyl alcohol. Following a final centrifugation, the pellet was thoroughly mixed in a tiny volume of 50% alcohol, taken up into a Pasteur pipette, and slowly dispensed dropwise on the surface of a stack of fast filter papers (Whatman 41).
5. Between droplets, the liquid was absorbed by the filter paper. As a result, a "pile" of cellular material was formed on the surface of the filter paper, which could be removed with a fine spatula and deposited on a 2–4% agar plate. At 60 °C, the material was covered with molten agar.
6. Following the removal of extra agar surrounding the specimen's, the "block" of material was treated in the manner indicated by^52,53^ as follows: dehydrated using ascending grades of ethanol alcohols (70%, 90 p%, 100I%, 100II%) and embedded in Epon-araldite. The blocks were dehydrated using a mixture of ethanol and propylene oxide. Blocks were dehydrated in increasing degrees of ethanol for 30 min, 70 min overnight, 90 min, 100% I for 30 min, and 100% II for 60 min.
  Resin embedding was carried out using propylene oxide (Merck, Darmstadt, Germany) for 30 min, Epon : propylene oxide (approximately 1:1 ratio) for 30 min, and Epon for 3 h. Epon was generated by combining 5 mL Epon 812 (Polysciences, Eppelheim, Germany) with 5 mL Araldite and 12 mL DDSA.
7. Epon was used to implant the blocks, which were then incubated at 60 °C. An Epon mix and an accelerator (DMP-30) were used to polymerize the samples (1.5%). The blocks were incubated for 3 days at the following temperatures: 60 °C on day one, 70 °C on day two, and 75 °C on day three.
8. Toluidine blue was used to stain semi-thin sections (1 micron) cut using an ultra-microtome Ultra cut E (Reichert Leica) (sodium tetra borate [borax] 1 g, toluidine blue 1 g, and distilled water 100 ml)^54^. The stained sections were examined using a Leitz Dialux 100 microscope. Photographs were taken with a Canon digital camera (Canon Power shot A95).

### Ultrathin section preparations for transmission electron microscope preparation (TEM)

We chose portions with sperm bundles from semi-thin sections to make ultrathin sections. An Ultrotom^®^ V was used to cut ultrathin portions (LKB Bromma, Munich, Germany). The 70 nm sections were stained with uranyl acetate and lead citrate^55^ and studied using a JEOL100CX II transmission electron microscope at Assiut University's Electron Microscopy Unit in Assiut, Egypt.

### Scanning electron microscope preparation (SEM)

The development of sperm bundles was observed using a scanning electron microscope (SEM). Sample preparation was carried out in accordance with^56^ as follows: Each sample was fixed in Karnovsky fixative (Table 2)^46^ for 4 h at 4 °C. The samples were then centrifuged and washed in the same fixation buffer (5 times) before being post fixed for 2 h at room temperature in 1% osmic acid in 0.1 M Na phosphate buffer. They were rinsed 15 times with 0.1 M Phosphate buffer. Ascending grades of ethanol alcohol with concentrations of 50, 70, 90, and 100 percent were used to dry the samples (30 min in each concentration). Each sample was distributed on an aluminum foil boat, fastened, and then air-dried. Finally, the samples were coated with gold using a JEOL1100 E ion sputtering device and examined at KV10 using a JEOL scanning electron microscope (JSM5400 LV).

**Table 2:** The swimming velocity and the percentage of sperm showing positive rheotaxis in lonesome sperm and thread-like bundles.

Types of sperm motility	Positive rheotaxis (%)	VCL (µm/s)	VAP (µm/s)	VSL (µm/s)
Lonesome sperm	52.3 ± 3.5	68.1 ± 0.8***	65.02 ± 0.6***	57.9 ± 0.7***
Thread-like bundles	85.2 ± 2.1**	31.3 ± 0.7	31.2 ± 0.4	26.3 ± 0.6

curvilinear VCL, straight-line VSL, and average path velocity VAP.

(means ± SD) was used to express all measurements.

*Significant at (P ≤ 0.05); **Significant at (P ≤ 0.01); ***Significant at (P ≤ 0.001).

### Digital coloring of scanning and transmission electron microscopic images

To detect the precise structure, we digitally colored the scanning and transmission electron microscopic pictures in Photoshop. Many authors have already employed this methodology^55–62^.

### Statistical analysis

All data were expressed as the mean ± SD. Kruskal–Wallis ANOVA on ranks was used to compare mean values, followed by Dunn's multiple comparisons. All statistics were calculated with the help of either JMP v5.0.1 (SAS campus drive, Cary, NC, USA) or GraphPad Prism v5 software (GraphPad Software, Inc., San Diego, CA). Differences of P < 0.05 were regarded as significant.

We utilized Kruskal–Wallis ANOVA to compare the lone sperm and bundle motions, which had various sample sizes. This method is used to compare two or more independent samples of equal or different sample sizes.

## Supplementary Information

Supplementary Legends.

Supplementary Video 1.

Supplementary Video 2.

Supplementary Video 3.

Supplementary Video 4.

Supplementary Video 5.

Supplementary Video 6.

Supplementary Video 7.

Supplementary Video 8.

## Data Availability

All data created or analyzed during this investigation are included in this published paper and are available on request from the authors.
