# Supplementary material for: New insights into sperm rheotaxis, agglutination and bundle formation in Sharkasi chickens based on an in vitro study
Source: Sci Rep. 2022 Jul 29;12:13003. doi: 10.1038/s41598-022-17037-x (PMC9338266; doi:10.1038/s41598-022-17037-x)
Supplement: Supplementary file 1 — Supplementary Legends. [file 41598_2022_17037_MOESM1_ESM.doc]

**Legend**

Videos:

**Video 1**: In a flowing fluid (velocity = 33 ± 5 µm/s), lonesome sperm rheotaxis behaviour is demonstrated. The rheotactic movement was described as sperm swimming against the direction of fluid flow. The direction of flow is indicated by the arrow.

**Video 2**: Showing a rheotactic sperm bundle (indicated by a red arrow) swimming against the flow direction (flow velocity = 33 ± 5 µm/s). The direction of flow is indicated by the arrow.

**Video 3**: Under a phase contrast microscope, video 3 shows some of the parallel thread-like bundles formed shortly after ejaculation.

**Video 4**: A video of a sperm bundle swimming in the microfluidic channel, taken by a 782 frame/sec high-speed camera, shows a developing bundle with the heads and mid-pieces of the front sperm being free and the tails and distal sperm in the terminal part of the bundle are agglutinated. The free heads are shown to be responsible for the movement by dragging the bundle.

**Video 5**: To avoid being carried away by the high-velocity flow, the free heads of two sperm bundles stuck strongly to the microchannel wall.

**Videos 6 and 7**: Several growing sperm bundles are capturing as many sperm as possible, swimming parallel to each other, intersecting and overlapping as an attempt to form bigger threads. The bundles eventually act as long threads, catching single sperm and small bundles. The lengthy threads of sperm began to form a net of sperm threads as the flow slows.

**Video 8**: Sperm bundle activity at high flow velocity (V >33 µm/s), where spiral thread movements accelerate as an attempt to adhere to other sperm bundles and to the microchannel sidewall.
